# Effects of Machining Parameters on Abrasive Flow Machining of Single Crystal γ-TiAl Alloy Based on Molecular Dynamics

**DOI:** 10.3390/mi16010084

**Published:** 2025-01-13

**Authors:** Junye Li, Chao Song, Xin Du, Hongcai Xie, Jinghe Zhao, Ying Chen

**Affiliations:** 1 Ministry of Education Key Laboratory for Cross-Scale Micro and Nano Manufacturing, Changchun University of Science and Technology, Changchun 130022, China; ljy@cust.edu.cn (J.L.); songchao1234s@163.com (C.S.);; 2 School of Mechanical Engineering, Changchun Guanghua University, Changchun 130033, China; 3 Jilin Province Product Quality Supervision and Inspection Institute, Changchun 130103, China

**Keywords:** machining parameters, molecular dynamics, abrasive flow machining, single crystal γ-TiAl alloy

## Abstract

Observing the intricate microstructure changes in abrasive flow machining with traditional experimental methods is difficult. Molecular dynamics simulations are used to look at the process of abrasive flow processing from a microscopic scale in this work. A molecular dynamics model for micro-cutting a single crystal γ-TiAl alloy with a rough surface in a fluid medium environment is constructed, which is more realistic. The evolution of material removal, cutting force, temperature, energy, and dislocation during micro-cutting are analyzed. The impact of cutting depth, abrasive particle sizes, and abrasive material on the micro-cutting process are analyzed. The analysis shows that the smaller cutting depth and abrasive particle sizes are beneficial to obtain a better machining surface, and the cubic boron nitride (CBN) abrasive is an effective substitute material for diamonds. The purpose of this study is to provide unique insights for improving the material removal rate and subsurface quality by adjusting machining parameters in actual abrasive flow precision machining.

## 1. Introduction

As a new type of lightweight high temperature structural material, γ-TiAl alloy is considered an excellent candidate material for high temperature applications and is highly favored in the fields of aerospace and automotive manufacturing. However, its low room temperature plasticity, poor fracture toughness, and difficult processing limit its wide application. As a micro machining technology, abrasive flow machining can achieve plastic removal of brittle materials. Therefore, studying the plastic deformation behavior and material removal characteristics of γ-TiAl alloy materials during abrasive flow machining is of great practical significance. However, the process of abrasive micro-cutting is a dynamic process at the microscale, and it is difficult to observe micro details in real-time using traditional experimental methods. This article uses a molecular dynamics simulation method to study the abrasive flow machining process at the microscale, revealing the mechanical behavior and micro-cutting characteristics of abrasive flow machining of γ-TiAl alloy materials. In abrasive flow precision machining, abrasive particles function as cutting tools. These particles repeatedly collide with and cut the surface of the workpiece, achieving the goal of finishing the piece. However, the micro-cutting process of abrasive flow only occurs in local areas of the machined surface. Directly observing material removal and deformation in the machining process through experimental methods is difficult due to the microscopic nature of material removal in the cutting area. Therefore, using molecular dynamics simulation to analyze micro-cutting characteristics in abrasive flow machining is essential to studying abrasive flow precision machining process [1,2,3,4].

In the actual machining process, the importance of abrasive flow machining parameters on machining performance or quality cannot be ignored. Appropriate parameters such as abrasive type, particle size, concentration, and flow rate can significantly improve material removal efficiency, shorten processing time, and ensure that the surface roughness and processing accuracy of the workpiece meet the expected requirements. The optimization selection of these parameters is crucial for achieving efficient and high-quality machining results. Therefore, in practical applications, it is necessary to fully understand and master the impact of various processing parameters on processing quality, so as to make precise adjustments according to specific needs and improve the overall processing level. Zhao et al. investigated the influence mechanism of coupling effects on the removal process of 3C-SiC materials under nanoscale conditions. The research showed that different grain shapes generate different grinding forces at the same preset depth and grinding speed, determining the surface processing quality [5]. Wang et al. investigated the impact of cutting depth on nano abrasive machining of GaN. The research results indicated that a larger cutting depth would promote an increase in cutting force, stress, and temperature, as well as the nucleation and expansion of dislocations and the degree of deformation of the subsurface layer [6]. Guo et al. conducted a study on the impact of different crystal orientations on the nano-cutting of single crystal tungsten. Their analysis of changes in crystal structure and the evolution of dislocations revealed that processing along the <110> crystal direction results in the best surface quality and the most minor depth of the subsurface deformation layer [7]. Li et al. systematically studied the mechanism of subsurface damage and material removal of nanoscale processed single crystal copper. The study analyzed the impact of cutting depth, abrasive particle sizes, and crystal orientation on material removal and workpiece deformation. The findings indicate that a smaller cutting depth and abrasive particle sizes can reduce the damage to the subsurface and improve the quality of the machined surface. Additionally, processing along the (001)[110] crystal direction was found to reduce surface roughness and enhance the quality of the surface [8]. Goel et al. conducted a study on the utilization of CBN tools instead of diamonds for the nanoscale processing of single crystal silicon because of the disadvantages of the high cost of diamonds. The research results indicated that CBN tools benefit the nanofabrication of single crystal silicon [9]. Li et al. used MD simulations to study the abrasive flow processing of different polycrystalline materials and the impact of cutting depth and abrasive particle types on the machining process. It was found that the different cutting depths and abrasive particle materials would produce different processing effects [10]. These studies provide an important reference for studying the nano-machined rough surface single crystal γ-TiAl alloy with varying machining parameters in the fluid medium environment.

The γ-TiAl alloy is an emerging high temperature structural material with strong development potential, characterized by low density, high stiffness, and good oxidation resistance. It is suitable for aerospace and automotive manufacturing fields. Previous research has only focused on studying gamma TiAl alloys with smooth surfaces in vacuum environments. Considering that the actual abrasive flow machining process is not a vacuum machining environment, but rather achieves finishing machining in fluid media such as hydraulic oil or aviation kerosene, the actual machined part surface is not completely smooth. Therefore, it is necessary to conduct research on the machining process of γ-TiAl alloys with rough surfaces in fluid media environments. Cao et al. investigated the effects of grain size on the atomic plastic flow mechanism, average cutting force, and stress variation in polycrystalline γ-TiAl alloy during nano-cutting using molecular dynamics methods [11]. Yao et al. investigated the micro defects and residual stress distribution of single crystal γ-TiAl during nano-cutting and annealing processes using molecular dynamics methods [12]. Feng et al. studied the material removal mechanism of single crystal γ-TiAl during nano-cutting using molecular dynamics methods, distinguishing between nano-cutting and macro-cutting [13]. These studies have produced significant research findings and provided valuable insights into the nanoscale processing of γ-TiAl alloys.

At present, many scholars have carried out research on nanoscale processing involving medium environments and workpieces with rough surfaces. Molecular dynamics methods not only enable real-time observation of atomic motion, but also collect valuable microscopic dynamic information, making them an effective experimental supplement. With the rapid increase in computer processing speed, many researchers are using molecular dynamics methods to study processes such as cutting, wear, friction, tension/shear, scratching, and indentation at the microscale. Yang et al. conducted the first study on the nanowear mechanism of KDP using molecular dynamics (MD) analysis. Nano scratch simulations were conducted at different scratch depths in various crystallographic directions, revealing the deformation and material removal mechanisms of KDP crystals, and establishing scratch maps that minimize subsurface damage in KDP crystals. Research has found that the deformation mechanism of KDP under nanowear is greatly influenced by material anisotropy. The size of the indenter and the depth of scratches have a significant impact on the surface integrity of machined KDP components [14,15]. Wu et al. used large-scale molecular dynamics simulations to investigate the influence of crystal position modes on the deformation of single crystal 6H-SiC, and the effects of particle cutting depth and abrasive particle size on the nano grinding deformation and material removal mechanism of 6H-SiC. The research results revealed the plastic deformation mechanism of single crystal 6H-SiC under different crystal position modes. The correct parameter selection (particle cutting depth and abrasive particle size) can significantly improve material removal efficiency and surface quality, which is the key to achieving high-quality processing [16,17]. Li et al. investigated the nanoscale processing of single crystal Cu with rough surface by MD simulation [18]. Wang et al. conducted a study to investigate the impact of the fluid medium on material removal and subsurface defect evolution of single crystal copper through molecular dynamics simulation [19]. Song et al. analyzed the surface material removal and damage mechanisms of silicon and amorphous silica in aqueous environments using molecular dynamics methods [20]. Li et al. investigated the abrasive flow machining mechanism of three polycrystalline materials (polycrystalline Cu, polycrystalline Fe, and polycrystalline Al) in a hydraulic oil environment by MD simulation [10]. Tong et al. studied the effect of textured surfaces on friction performance in nanoscale rolling contact using molecular dynamics simulations. The results indicate that textured surfaces can effectively improve friction characteristics, verifying the rationality of using textured surfaces in simulations [21]. Hinkle et al. studied the formation of self-affine roughness on the surface of homogeneous materials during deformation through molecular dynamics simulations, revealing the origin of surface roughness [22]. Zhang et al. provided a comprehensive overview of the latest developments in molecular dynamics simulations of dry friction on rough substrates, discussing various methods for constructing rough surfaces, including grooved textured surfaces, fractal surfaces, Gaussian surfaces, and their applications in simulations [23]. These studies provide an important reference for studying nanoscale processing of single crystal γ-TiAl alloys with rough surfaces.

In the course of this study, molecular dynamics methods were used to study the micro-cutting process of a single crystal γ-TiAl alloy with rough surfaces in a fluid medium environment. The impact of different machining parameters (cutting depth, abrasive particle size, and abrasive material) on material removal, cutting force, temperature, energy, and dislocation evolution during micro-cutting were analyzed. The analysis reveals the mechanical behavior and micro-cutting characteristics of γ-TiAl alloy material processed by abrasive flow processing from a microscopic point of view. The aim is to provide unique insights for increasing the rate of material removal and improving the subsurface quality of the actual abrasive flow precision machining by adjusting the processing parameters and promoting the development of abrasive flow machining technology.

## 2. Build Model and Select Potential Functions

### 2.1. Build the Model

**Figure 1:**
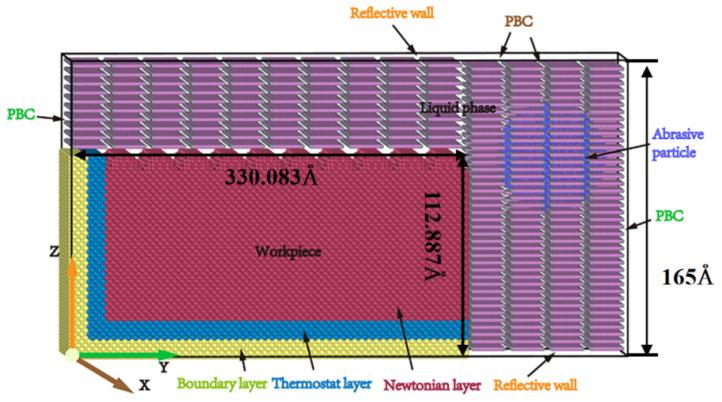
Micro-cutting model of rough surface of γ-TiAl alloy in fluid environment.

This study selects a single crystal γ-TiAl alloy as the workpiece material, and MD simulation is performed by LAMMPS (64-bit 28Jul2021-MPI) Molecular Dynamics Simulator (LAMMPS) software [24]. The OVITO (3.6.0)software can observe changes in the MD model during nano-cutting simulation [25]. The molecular model of the fluid medium is constructed using Moltemplate software. An L10 face-centered tetragonal (FCT) structure makes up the crystal structure of the γ-TiAl metal. The lattice constants are a = b = 4.001 Å and c = 4.181 Å. The crystal structure is like the face-centered cubic (FCC) structure, except that at the center of the four side faces are aluminum atoms instead of titanium atoms. A molecular dynamics model of a single crystal γ-TiAl alloy workpiece was established, with a uniformly rough surface, as shown in Figure 1. In the long-term research process, it was proven that using textured surfaces instead of special rough surfaces is a simple and efficient research method [18]. Therefore, in this study, textured surfaces are used instead of rough surfaces. 

A single crystal γ-TiAl alloy model was established using a three-dimensional coordinate system. The dimensions of the model in the crystal directions of [100], [010], and [001] were 120.03 Å × 330.083 Å × 112.887 Å, respectively, containing 258,446 Ti and Al atoms. The abrasive particles are diamond abrasive particles with a radius of 3 nm and containing 19,884 atoms. Diamond particles are selected as the abrasive particles, and the model has a size radius of 3 nm and contains 19,884 C atoms. During the simulation, these diamond abrasive particles are treated as rigid bodies due to their significantly greater hardness compared to the single crystal γ-TiAl alloy. The abrasive particles are situated 2 nm away from the right side of the workpiece, in the upper right corner. The liquid phase consists of n-dodecane C_12_H_26_ molecules totaling 7851 in number. Alkane molecules utilize the united atom model where CH_3_ and CH_2_ groups are considered a single point of interaction. The TraPPE-UA force field is employed to describe intermolecular interactions. The simulation process is divided into the relaxation phase and the processing phase. To minimize the energy of the system as much as possible, the conjugate gradient method was used. Afterward, use the Nose Hoover hot bath method to maintain the temperature settings of the fluid medium and thermostat layer at 293 K. When the potential energy and temperature reach an equilibrium stable state, it proves that the relaxation stage has ended. Then, enter the processing stage using NVE integrated processing; the above temperature is still maintained at 293 K. After the relaxation process is completed, set the speed of the abrasive particles to 100 m/s and move in the -Y direction. Table 1 shows the specific conditions and parameters used in the simulation.

Cubic boron nitride (CBN), cubic silicon carbide (SiC), and diamond are three common superhard materials with significant differences in mechanical properties, crystal structure, shape, and hardness distribution, and are commonly used as abrasives for abrasive flow machining. Silicon carbide has a diamond structure, which can maintain high strength at higher temperatures and significantly improve the surface quality of workpieces. Compared to diamond and CBN, although there is no significant difference in hardness of silicon carbide (Mohs hardness of 9–9.5), the fluctuation of hardness distribution may easily lead to uneven abrasive wear during processing. Its shape is mostly irregular granular, with significant friction and grinding effects, and its price is relatively cheap. Diamond is composed of carbon atoms arranged in a face-centered cubic lattice structure, with a lattice constant of 3.57 Å. It has a sharp crystal shape and extremely high hardness (Mohs hardness 10). It is precisely due to the existence of C-C covalent bonds that diamond exhibits excellent chemical stability, enabling it to achieve large cutting depths and efficient cutting performance during processing, and is widely used in the fields of precision and ultra precision machining. In contrast, cubic boron nitride (CBN) is composed of boron and nitrogen atoms, forming a sphalerite structure in the form of sp3 hybridization. Its shape is relatively regular, with dull edges, suitable for stable cutting, and its hardness is second only to diamond (Mohs hardness of about 9.5). It also has significant thermal stability and cost advantages, making it particularly suitable for processing iron-based materials under high temperature conditions. Therefore, in actual processing, the crystal structure, shape, and hardness distribution of different abrasives have a significant impact on the material removal mechanism and processing effect. When selecting abrasives, it is necessary to consider these factors comprehensively in order to achieve efficient processing, reduce surface defects, and improve surface quality.

### 2.2. Select Potential Functions and Relevant Parameters

#### 2.2.1. EAM Potential Function

EAM is utilized to characterize the interaction forces among metal atoms. In this study, EAM is employed to delineate the interaction potential between Ti-Al alloys [26] and is expressed as Equation (1) [27]. The parameters used in the simulation process are from Wang [28].

(1) $$E=\underset{i}{\sum }F_{i}\left(\rho_{i}\right)+\frac{1}{2}\underset{i\neq j}{\sum }\emptyset_{ij\left(r_{ij}\right)}$$

Here, $F_{i}$ represents the embedded energy function of atom *i*, and $\phi_{ij}$ represents the potential interaction function between atoms *i* and *j*.

#### 2.2.2. Morse Potential Function

The Morse potential function is utilized to characterize the interaction between abrasive particles and the workpiece being processed [29,30,31]. In this study, the Morse potential is employed to describe the interaction between Ti-C, Ti-B, Ti-N, Ti-Si, Al-C, Al-B, Al-N, and Al-Si [32], expressed as Equation (2). Morse potential function parameters are shown in Table 2 [33,34,35].

(2) $$E=D\left[e^{-2\alpha \left(r_{ij}-r_{0}\right)}-{2e}^{-\alpha \left(r_{ij}-r_{0}\right)}\right]$$

Here, D represents the binding energy, α represents the Modulus of elasticity, and $r_{0}$ represents the distance between atoms at equilibrium.

#### 2.2.3. Lennard-Jones Potential Function

The L-J potential function is utilized to depict the interactions among molecules in liquid substances. In this study, the united atom (UA) model is used to describe alkane molecules, utilizing the TraPPE-UA force field [35] to describe intermolecular interactions. The CH_2_ and CH_3_ groups are considered single interaction points, and the hydrogen atoms contained within them only show the mass in the united atom mode for faster and more accurate calculations [36]. The expression of its potential function is Equation (3). Table 3 shows L-J potential parameters [37,38].

(3) $$U_{L-J}=4\varepsilon \left[{\left(\frac{\sigma }{r}\right)}^{12}-{\left(\frac{\sigma }{r}\right)}^{6}\right]$$

Here, σ represents the equilibrium distance between atoms when the potential is 0, and ε represents the depth of the potential well, reflecting the strength of the mutual attraction between two atoms. 

## 3. Results and Discussion

### 3.1. Distribution Analysis of Fluid Medium

**Figure 2:**
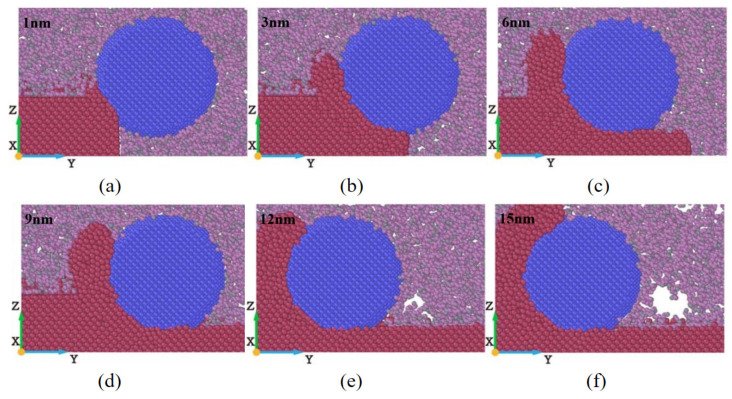
Transient distribution cross-sectional diagram of fluid medium during micro-cutting process. Displacement distances of cutting particles are (**a**) 1 nm, (**b**) 3 nm, (**c**) 6 nm, (**d**) 9 nm, (**e**) 12 nm, and (**f**) 15 nm.

After the relaxation stage is completed, fluid molecules will adsorb onto the surface of the workpiece and abrasive particles. When the abrasive particles move along the machining direction, the fluid molecules are also subjected to the squeezing effect of the abrasive particles, causing the fluid to flow around the workpiece and the surface of the abrasive particles. In order to study the distribution of the fluid medium during the machining process, instantaneous distribution cross-sections of the fluid medium at different displacement distances of the cutting particles were measured, as shown in Figure 2.

As is well-known, the main functions of fluids are twofold: firstly, to achieve lubrication by reducing the friction generated in the contact area between abrasive particles and the machined surface of the workpiece, as well as between abrasive particles and chips; secondly, to achieve cooling and heat dissipation by adsorbing on the surface of the workpiece. The combined effect of lubrication and cooling improves the quality and machining accuracy of the machined surface. From Figure 2, it can be seen that there are almost no fluid molecules present in the contact area between the abrasive particles and the workpiece. This is because the stress in the contact area between the abrasive particles and the workpiece is relatively high, which is not conducive to the infiltration of fluid molecules into it. In the actual machining process, the fluid in the contact area between the abrasive particles and the workpiece usually forms a lubricating oil film, which can reduce friction and cooling, thereby affecting the material removal process. It can be noted that some fluid molecules are adsorbed on the chips and processed surfaces. This is because the adsorption energy between fluid molecules and the surface of the workpiece is much greater than the interaction energy between fluid molecules. Fluid molecules can easily break through the hindrance of intermolecular forces and produce displacement, and ultimately adsorb onto the surface of the workpiece. In addition, the intermolecular forces between alkane molecules can give the fluid molecular layer a certain strength, which promotes the stable formation of the lubricating oil film, thereby achieving the effect of reducing friction and anti-wear. Although some fluid molecules may move and diffuse to the surface of the workpiece and adsorb onto it, they do not diffuse to the contact area between the abrasive particles and the workpiece from the beginning of the machining process. This may be due to weak interactions between the fluid and the workpiece, as well as between the fluid and the abrasive particles. However, the wetting characteristics of fluids play a crucial role in the contact process between abrasive particles and workpieces. Additionally, the high-pressure barrier effect in the contact zone prevents fluid molecules from infiltrating, as their kinetic energy is insufficient to overcome the compressive forces. Despite this limitation, the adsorption and redistribution of fluid molecules at the boundary regions can stabilize lubrication and improve machining accuracy. Furthermore, molecular dynamics simulations have shown that increasing the adsorption energy of fluid molecules significantly enhances their ability to form a continuous lubricating film, reducing friction and heat generation. Studies suggest that fluid molecules with higher polarity or longer molecular chains are more effective in forming stable lubricating films. For instance, alkane-based fluids with long chains exhibit enhanced intermolecular forces that improve their ability to adhere to both the abrasive particles and the workpiece surface. Additionally, fluid diffusion around the contact area facilitates the removal of micro-debris, further contributing to smoother surface finishes. By interacting with high energy regions on the workpiece surface, the fluid molecules also aid in dissipating localized heat, reducing the risk of thermal damage. As the displacement distance of cutting particles increases, the fluid molecules in front of the abrasive particles are squeezed by the abrasive particles, and it is difficult for them to quickly flow behind the abrasive particles due to the obstruction of chips. The fluid molecules behind the abrasive particles will diffuse and move along the machining direction with the abrasive particles, resulting in the gradual formation of voids on the machined surface of the workpiece, as shown in Figure 2e,f. The interaction of fluid molecules with the chips and processed surface highlights the dynamic nature of lubrication during machining, emphasizing the importance of selecting fluid media with optimal physical and chemical properties to enhance overall performance.

### 3.2. Material Removal and Surface Formation Analysis 

The atoms in the γ-TiAl alloy workpiece are arranged in space in a regular and orderly way when there is no outside force acting on them. Due to the abrasive particles moving in a cutting direction, the front end atoms of the workpiece surface are subjected to shear and extrusion, and the surface of the workpiece will be deformed. From a microscopic point of view, the atoms on the surface of the workpiece will be displaced and gathered at the front end of the workpiece surface and abrasive particles. In this study, the material removal and surface formation process of the γ-TiAl alloy workpiece surface under the different displacement distance of cutting particles, cutting depths, abrasive particle sizes, and abrasive materials were studied, and surface morphology maps were obtained under different processing parameters.

**Figure 3:**
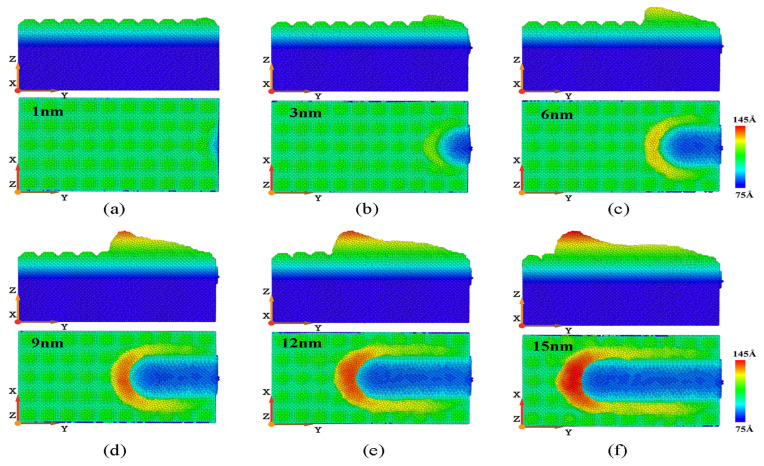
Cross-sectional view and surface morphology of γ-TiAl alloy workpiece during processing. Displacement distances of cutting particles are (**a**) 1 nm, (**b**) 3 nm, (**c**) 6 nm, (**d**) 9 nm, (**e**) 12 nm, and (**f**) 15 nm. (Coloring of workpiece atoms is based on their height and position on Z-axis. Except for workpiece atoms, other abrasive particle atoms and fluid medium atoms are not studied and considered).

The workpiece’s cross-sectional view and surface morphology at different displacement distances of cutting particles are shown in Figure 3. Figure 3a shows that when abrasive particles begin to come into contact with the workpiece, the atoms on the surface of the workpiece are squeezed and begin to shift. Most of the workpiece atoms are displaced towards the front and bottom of the abrasive grain under compression, while there are relatively fewer atoms towards the front and top. Figure 3b shows that the displacement distances of the cutting particles continually increases, and the cutting force gradually increases along with the displacement distance of cutting particles. When the normal cutting force increases to a certain extent, the workpiece atoms gradually begin to move in the normal direction, and more workpiece atoms are gathered at the front end of the abrasive particles, gradually forming chips. The displacement distance of the cutting particles shown in Figure 3c is 6 nm, more workpiece atoms are displaced to the front and above the abrasive particles, and the displacement is more obvious. In addition, the accumulation of a large number of workpiece atoms at the front end of the abrasive particles hinders the cutting motion of the abrasive particles. The squeezing effect of the abrasive particle edge causes the workpiece atoms in the chip area and the area below it to move to both sides of the abrasive particles, causing sidestream atoms to appear on both sides of the groove. Therefore, more accumulated atoms in front of the abrasive particles flow to both sides of the groove. With the continuous cutting motion of the abrasive particles, the displacement distance of the cutting particles continues to increase, and more and more workpiece atoms are displaced, accumulating at the front end of the abrasive particles. One part is removed in the form of chips, and the other part stays on both sides of the groove in the form of side flow.

**Figure 4:**
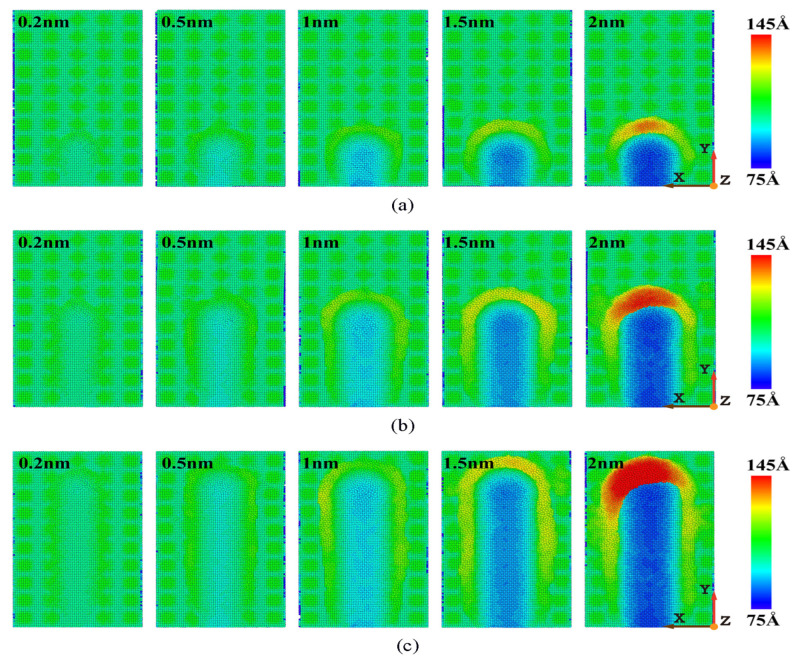
Variation in surface morphology with cutting depths of 0.2 nm, 0.5 nm, 1 nm, 1.5 nm, and 2 nm. (Displacement distances of cutting particles are (**a**) 6 nm, (**b**) 12 nm, and (**c**) 18 nm).

The shape of the workpiece’s morphology at different cutting depths is shown in Figure 4. Figure 4 shows the factors that have a greater impact on cutting depth on machining and material removal. Under the same displacement distance of cutting particles, the deeper the cutting depth, the more displaced the workpiece atoms, that is, more and more atoms are removed. This is because when the displacement distance of the cutting particles increases, the abrasive particles can come into contact with more atoms inside the workpiece, which causes more workpiece atoms to shift and move to the front and above the abrasive particles, forming more chip atoms. In addition, in the cutting process, the part of the atoms will also be squeezed and forced to flow to both sides of the groove when in contact with abrasive particles, forming side flow. At different cutting depths, the amount of material accumulation on the side of the corresponding groove varies greatly. As the cutting depth increases, the number of workpiece atoms flowing to both sides of the groove due to the sidestream is also increasing. As shown in Figure 4c, after the machining process, the maximum number of material removal atoms is observed when the cutting depth is 2 nm, but the corresponding number of sid flow atoms is also the highest, which indicates that a high chip thickness can greatly increase the number of side flow atoms.

**Figure 5:**
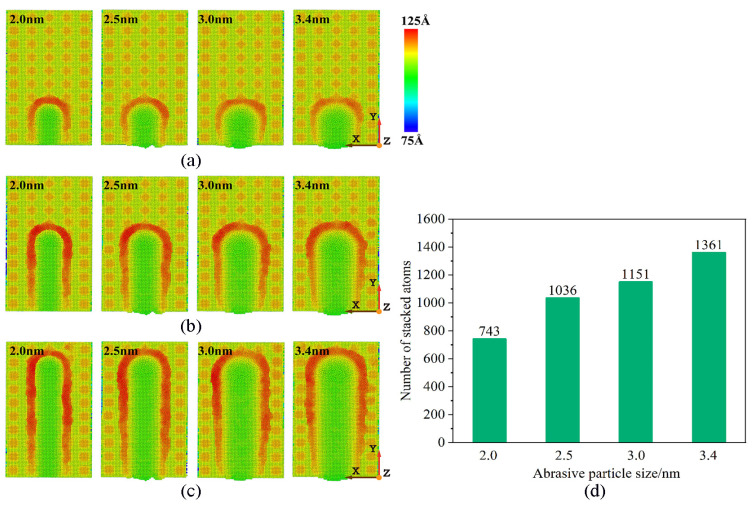
Variation in surface morphology with abrasive particle sizes of 2.0 nm, 2.5 nm, 3 nm, and 3.4 nm. (Displacement distances of cutting particles are (**a**) 6 nm, (**b**) 12 nm, and (**c**) 18 nm.) (**d**) Relationship between number of stacked atoms and abrasive particle sizes.

The surface morphologies of workpieces’ abrasive particles of different sizes is shown in Figure 5. Figure 5 shows that the atoms of the workpiece are squeezed, causing them to accumulate at the front of the abrasive atoms and on both sides of the groove. With the size of the abrasive particles increasing, more workpiece atoms accumulate at the front of the abrasive particles. This is because the larger the abrasive particles, the more workpiece atoms come into contact with the abrasive particles thereby increasing the cutting range of the abrasive particles and enhancing the cutting effect. In the X direction, more workpiece atoms will shift, causing the chip atoms formed by cutting to gather more evenly in front of the abrasive particles. Therefore, a larger abrasive particle size will increase the number of chip atoms. In addition, in the actual processing and production process, the larger the size of abrasive particles, the greater the initial cutting depth. Therefore, with the continuous increase in displacement distance of cutting particles, the influence of abrasive particle size is more significant compared to other processing parameters in the cutting process. However, during the machining process, it is not necessarily better to have larger abrasive particle sizes, as material properties need to be considered. At room temperature, γ-TiAl alloy is a brittle material, and when selecting particle size, it is necessary to consider that the cutting depth should not exceed the critical value of the material’s ductile–brittle transition depth. Therefore, we should choose the abrasive particle sizes reasonably based on increasing the material removal rate as much as possible.

**Figure 6:**
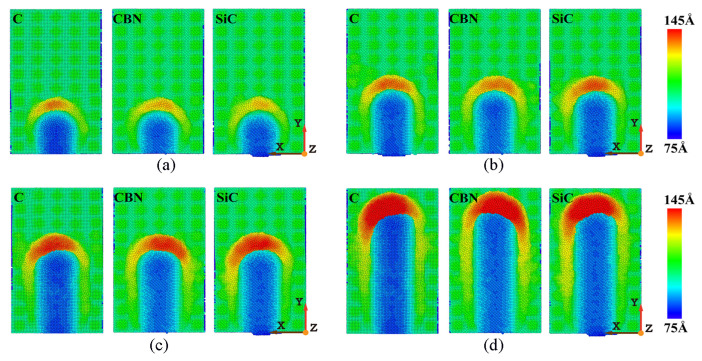
Variation in surface morphology with abrasive materials of diamond (C), cubic boron nitride (CBN), and silicon carbide (SiC). (Displacement distances of cutting particles are (**a**) 6 nm, (**b**) 9 nm, (**c**) 12 nm, and (**d**) 18 nm).

The surface morphology of workpieces with abrasive particles of different materials is shown in Figure 6. The figure shows that there is not much difference in the number of atoms deposited in the front when three different abrasives are selected to process the γ-TiAl alloy workpiece. It can be clearly seen that the chip shapes of γ-TiAl alloy workpieces processed by different abrasive materials are different. A large number of chips accumulate on the left front of the abrasive particles when diamond abrasive particles and SiC abrasive particles are selected for processing. However, the chips will accumulate on the right front of the abrasive particles when CBN abrasive particles are selected. This is mainly because diamond has extremely high hardness and sharp and regular crystal surfaces, which can effectively tear materials. The cutting force is mainly concentrated in the front, and debris flows to the left front along the cutting direction. Silicon carbide has a high hardness but a hexagonal crystal structure, which tends to be cut through brittle fracture, causing debris to accumulate towards the left front along the angle of abrasive feeding. Although cubic boron nitride has lower hardness than diamond, it has higher thermal and chemical stability, especially at high temperatures. The cutting behavior of CBN can cause debris to accumulate in the front right, which is closely related to its excellent thermal stability and high friction coefficient. CBN effectively disperses cutting heat under high temperature conditions, while its crystal structure changes the force balance in the cutting area, making it easier for debris to accumulate to the front right. Because the actual machining process is multiple abrasive particles acting on the workpiece, the side flow phenomenon has little influence on the machined surface. Considering the high cost of diamonds and the comparable removal efficiency of the three types of abrasives, CBN abrasive particles or SiC abrasive particles can be chosen based on cost and processing efficiency considerations. 

### 3.3. Analysis of Cutting Force Changes

Due to the presence of cutting force, squeeze and shear occur between the abrasive particles and workpiece material, resulting in the cutting and removal of the material on the workpiece surface, achieving the machining process. Therefore, the magnitude of the cutting force can intuitively reflect cutting effectiveness. Because abrasive particles move in the -y direction, Fy is the main cutting force. The main cutting force Fy mainly causes displacement and accumulation of atoms on the workpiece surface, resulting in the formation of chips and lateral flow. The cutting force Fz primarily induces downward movement of the atoms located at the lower end of the abrasive particles, ultimately forming the machined surface. The cutting force Fx is the frictional force generated. 

**Figure 7:**
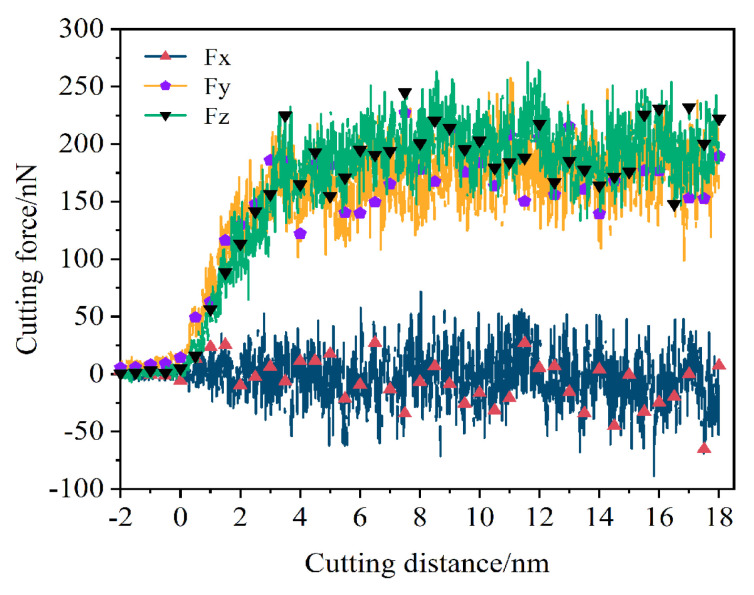
Variation in cutting force in each direction with increase in displacement distance of cutting particles in micro-cutting process.

The variation in cutting force at different displacement distances of the cutting particles is shown in Figure 7. The main cutting force shows a slow increase trend, but the increase trend is not obvious. The main cutting force Fy and the normal cutting force rise rapidly because the abrasive particles begin to act on the workpiece when the abrasive particles start to cut the workpiece. The lateral force Fx is mainly manifested as the frictional force between atoms. It fluctuates around Fx = 0 N during the whole process. According to the changing trend of cutting force, the machining process can be divided into an initial machining phase and a stable machining phase. In the initial processing stage, the main cutting force Fy and the normal force Fz have similar changing trends, both showing a rapid increase trend followed by a fluctuating upward and downward trend. From the trend of cutting force changes in the figure, it can be seen that cutting forces Fy and Fz play a decisive role in the final machining effect. The cutting forces Fy and Fz no longer continue to increase but start to fluctuate continuously around a certain value when the displacement distance of cutting particles reaches 3 nm. This shows that the processing stage has entered the stable processing stage from the initial processing stage. 

**Figure 8:**
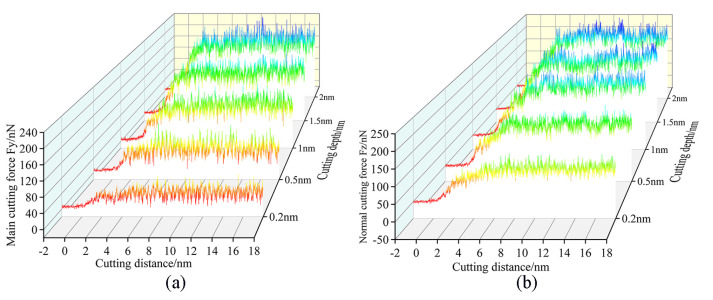
Variation in (**a**) main cutting forces and (**b**) normal cutting forces with cutting depths of 0.2 nm, 0.5 nm, 1 nm, 1.5 nm, and 2 nm.

The variation law of cutting force with the increasing displacement distance of the cutting particles at different cutting depths is shown in Figure 8. It can be seen that as the cutting depth increases from 0.2 nm to 2 nm, the cutting forces Fy and Fz show an increasing trend. This is because the contact area between the abrasive particles and the workpiece increases with the increase in cutting depth, and more workpiece atoms are sheared and squeezed by the abrasive particles, resulting in more atom displacement and accumulation, which requires abrasive particles to have greater cutting force. As the cutting depth increases, the displacement distance of the cutting particles at which the cutting force reaches a stable fluctuation also increases, which means that the workpiece requires a longer displacement distance of cutting particles to enter the stable cutting stage. In addition, the fluctuation range of different cutting depths is not the same, and the fluctuation range is wider with the increase in cutting depth. As mentioned earlier, the nucleation, expansion, and annihilation of the internal defect structure of the workpiece lead to the reciprocating fluctuation of the cutting force. Therefore, this progress can be explained as follows: the greater the cutting depth, the greater the cutting force acting on the workpiece atoms, and the greater the number of material atoms resulting in lattice deformation and lattice reconstruction, resulting in a phenomenon of different fluctuation amplitude of the cutting force.

**Figure 9:**
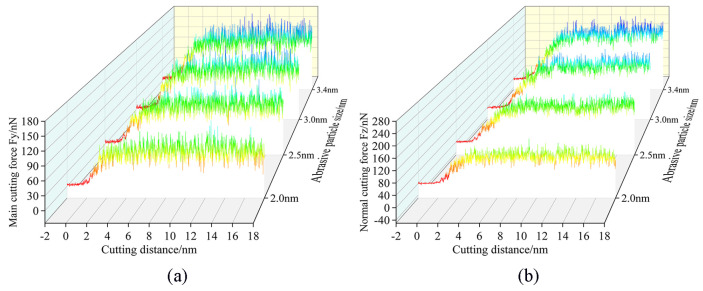
Variation in (**a**) main cutting forces and (**b**) normal cutting forces with abrasive particle sizes of 2.0 nm, 2.5 nm, 3.0 nm, and 3.4 nm.

The variation law of cutting forces with increasing displacement distances of the cutting particles at different abrasive particle sizes is shown in Figure 9. As the abrasive particle sizes increase from 2 nm to 3.4 nm, the cutting forces Fy and Fz show an increasing trend, and this trend becomes more obvious with the increase in the abrasive particle sizes. This can be explained as a larger abrasive particle size will increase the processing range, and more workpiece atoms will participate in the process; the abrasive particles will act on more of the workpiece atoms, and these workpiece atoms are displaced when subjected to squeeze and shear, moving in front of and above the abrasive particles to form chips, or moving under the abrasive particles to form the processed surface, which will require abrasive particles to provide greater cutting force.

**Figure 10:**
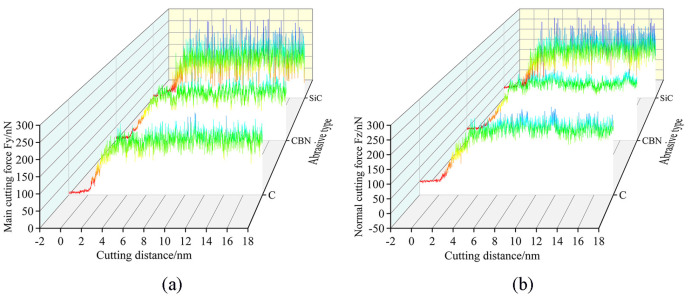
Variation in (**a**) main cutting forces and (**b**) normal cutting forces with abrasive materials of diamond (C), cubic boron nitride (CBN), and silicon carbide (SiC).

The variation law of cutting forces with increasing displacement distances of the cutting particles at different abrasive materials is shown in Figure 10. It is evident that the overall trends of the main cutting forces and normal cutting forces are relatively similar when machining the γ-TiAl alloy workpiece with three types of abrasives. However, when selecting the SiC grinding grain for processing workpieces, the phenomenon of fluctuating cutting force throughout the entire process is very apparent, with a relatively large range of fluctuations. As mentioned earlier, this fluctuation phenomenon is mainly related to the generation of lattice phase transformation, lattice reconstruction, the generation of amorphous structure, and the evolution of microstructure defects in the workpiece. This kind of fluctuation is the least obvious when choosing CBN abrasive particles to process the workpiece. It is somewhere along the lines of selecting diamond abrasive particles to process workpieces. Therefore, it can be considered that when selecting the SiC abrasive particle to process the γ-TiAl alloy workpiece, the lattice structure changes and defect structure activities inside the workpiece are more frequent, while the internal defect activity is less when choosing CBN abrasive particle processing. 

### 3.4. Temperature and Energy Change Analysis

Cutting heat is a crucial physical phenomenon in the process of machining. The machining process will be accompanied by the generation of cutting heat. A very small part of the energy consumed during processing is used to form a new surface and the storage of lattice deformation energy within the lattice, and most of the rest is converted into heat. Therefore, during processing, it can be considered that all energy is converted into heat, and the remaining energy can be ignored. A significant amount of heat generated from cutting processes can elevate the temperature of the workpiece, directly impacting the material’s performance, processing accuracy, and the quality of the finished surface. Therefore, the research and analysis of the temperature and energy change in the workpiece during the machining process are highly significant.

**Figure 11:**
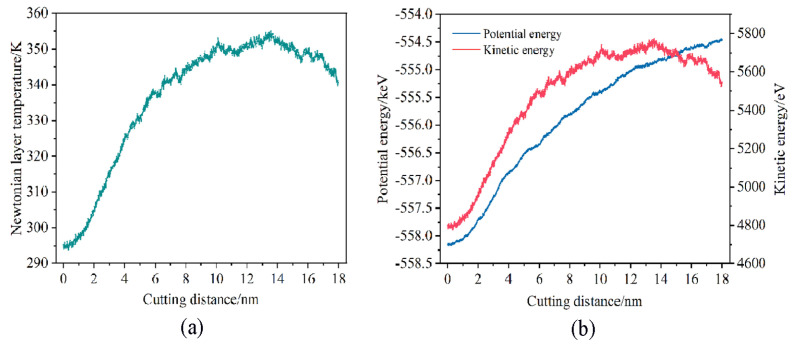
(**a**) The temperature and (**b**) energy changes in the workpiece during the micro-cutting process.

The variation law of the temperature and energy changes in the workpiece with the increasing displacement distances of the cutting particles is shown in Figure 11. The energy generated by the shear deformation of the chips and the friction between the abrasive particles and the workpiece is transformed into cutting heat, leading to an increase in temperature within the Newtonian layer of the workpiece. From Figure 11a, it is evident that when the displacement distance of the cutting particles is small, since the abrasive particles just start to contact the workpiece, only the squeezing effect is produced on the workpiece, the generated chips and lattice transformation are relatively small, and the cutting heat generated is less, so the temperature of the workpiece increases slowly. The temperature of the Newtonian layer of the workpiece increases with the increase in displacement distance of the cutting particles. On the one hand, the generation of a large amount of cutting heat is attributed to the workpiece atoms being squeezed by the abrasive particles, the chemical bonds between the workpiece atoms being destroyed, the workpiece atoms being displaced, and the kinetic energy of the atoms increasing. On the other hand, as the lattice strain energy stored in the crystal lattice is released, a portion of this energy is transformed into heat. When the displacement distance of cutting particles reaches 6 nm, the temperature of the workpiece Newtonian layer continues to show an increasing trend, but the increasing trend begins to slow down. This is due to the restructuring of the crystal lattice in the workpiece, and the combination of amorphous atoms consumes part of the energy. When the displacement distance of cutting particles exceeds 16 nm, there is a decreasing trend in the temperature of the workpiece’s Newtonian layer. On the one hand, this phenomenon is attributed to the consumption of lattice reconstruction and the combination of amorphous atoms. On the other hand, it is also due to the gradual approach of abrasive particles towards the thermostat layer, resulting in the quick transfer of cutting heat generated by the Newtonian layer to the thermostat layer thereby accelerating the heat balance of the workpiece. The entire process is accompanied by small temperature fluctuations, which are caused by the formation and evolution of the internal dislocation and defect structure of the workpiece. Generally, the increase in workpiece temperature will promote the evolution of internal defects, although the squeezing and shearing of abrasive particles still remain predominant.

From Figure 11b, it is evident that the atomic potential energy of the workpiece exhibits a gradual increase when the displacement distance of the cutting particles is small, albeit at a relatively slow pace. This is because the abrasive particles just start to contact the workpiece and only exert a squeezing effect on the workpiece, and the atomic displacement of the workpiece is small. As the displacement distance of the cutting particles increases, the potential energy of the workpiece atoms increases rapidly, accompanied by small fluctuations. This phenomenon is attributed to the movement of various dislocation defects during the cutting process. As the processing continues, the abrasive atoms interact with the workpiece atoms until the lattice strain energy stored in the atomic lattice of the workpiece exceeds the critical value of its atomic binding force hence the lattice structure will be destroyed and the workpiece atoms will produce displacement and misarrangement phenomenon. At this point, the strain energy is released, leading to a rapid increase in the potential energy of the workpiece. The change in the kinetic energy of the workpiece atoms reflects the work carried out by the abrasive particles on the workpiece atoms. To investigate the change in kinetic energy of the workpiece during the machining process, an analysis is conducted on the Newtonian layer’s kinetic energy variation with different displacement distances of the cutting particles. The kinetic energy $U_{k}$ of the workpiece atom i in the figure is calculated by Equation (4) according to the speed of a certain moment. The atoms of the workpiece are compressed and sheared by abrasive particles, causing an increase in the atomic temperature in the processing area, which intensifies the thermal motion of the workpiece atoms and generates displacement. At this time, the velocity $v_{i}$ is obtained in the direction of displacement, so the atomic kinetic energy in the machining processing area shows an increasing trend. Equation (5) shows the linear relationship between workpiece temperature and kinetic energy. It can be inferred from this that the change in kinetic energy closely mirrors the temperature changes.

(4) $$U_{k}=\frac{1}{2}\sum_{i-1}^{N}m_{i}(v_{ix}^{2}+v_{iy}^{2}+v_{iz}^{2})$$

Here, N represents the atomic number, $v_{ix}$ represents the speed in the x direction, $v_{iy}$ represents the speed in the y direction, and $v_{iz}$ represents the speed in the z direction.

(5) $$T=\frac{\sum_{i=1}^{N}m_{i}v_{i}^{2}}{3Nk_{B}}$$

Here, N represents the atomic number, $m_{i}$ represents the atomic mass, $k_{B}$ represents the Boltzmann constant, and $v_{i}$ represents the atomic velocity.

**Figure 12:**
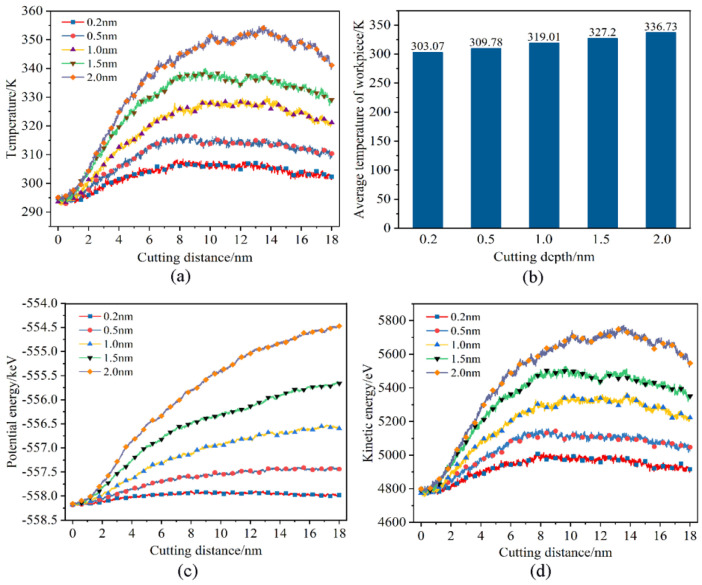
Effects of cutting depths. (**a**) Variation in Newtonian layer instantaneous temperature, (**b**) variation in Newtonian layer average temperature, (**c**) variation in potential energy, and (**d**) variation in kinetic energy.

The variation law of the temperature and energy changes in the workpiece with the increasing displacement distances of the cutting particles at different cutting depths is shown in Figure 12. It is evident that as the displacement distance of cutting particles increases, the temperature of the Newtonian layer of the workpieces with different cutting depths generally shows an increasing trend. It can be considered that when the heat dissipation efficiency is constant, as the displacement distance of cutting particles increases, the heat generated by the friction between the abrasive particles and the workpiece will increase accordingly; the efficiency of heat generation surpasses that of heat dissipation, and the temperature of the workpiece processing area will inevitably show an increasing trend. When the cutting depth is 0.2 nm, the temperature change curve of the workpiece is relatively flat, and the temperature increase rate is relatively slow. When the cutting depth increases, the temperature curve of the workpiece gradually becomes steeper, and the temperature rate gradually increases. This shows that the depth of cutting has a greater influence on the temperature of the workpiece. However, when the displacement distance of the cutting particles reaches a certain value, the temperature of the workpiece at various cutting depths exhibits a downward trend. The main reason is that the abrasive particles are gradually approaching the thermostat layer, and the cutting heat generated can be transferred to the thermostat layer faster; the heat dissipation efficiency is greater than the heat generation efficiency, which accelerates the heat balance of the workpiece. But the moment of temperature drop is quite different. It is seen that a smaller cutting depth results in relatively lower frictional heat, leading to the rapid establishment of a new equilibrium between heat generation and dissipation. Therefore, it can be inferred that the workpiece region’s temperature reaches a steady state relatively faster when the cutting depth is reduced. In addition, it can be seen from Figure 12b that as the cutting depth increases, the average temperature of the workpiece also increases. This is because as the depth of cutting increases, the contact area between the abrasive particles and the workpiece increases. This results in a simultaneous squeezing and shearing of more workpiece atoms by the abrasive particles, more displacement of workpiece atoms, and consequently generates more cutting heat. From Figure 12c,d, it is seen that as the cutting depth increases, the potential energy and kinetic energy of the workpiece show a gradually increasing trend. This is because as the depth of cutting increases, the contact area between the abrasive particles and the workpiece atoms increases, resulting in more atoms of the workpiece being subjected to the squeezing and shearing of the abrasive particles hence the energy generated by cutting increases, and then the potential energy and kinetic energy gradually increase. Combined with the analysis of the temperature change in the workpiece, it can be considered that a smaller cutting depth can reduce energy consumption through a relatively lower temperature. 

**Figure 13:**
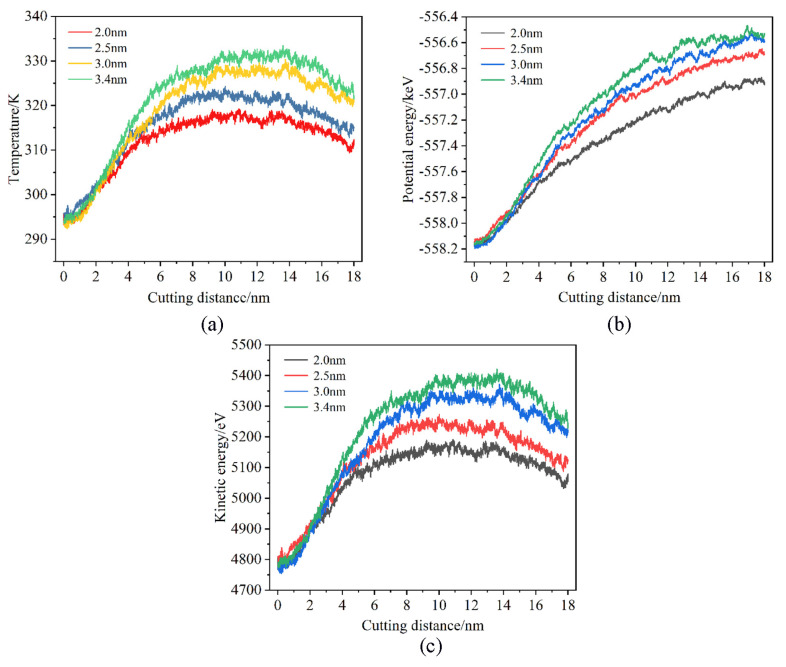
Effects of abrasive particle sizes. (**a**) Variation in Newtonian layer instantaneous temperature, (**b**) variation in potential energy, and (**c**) variation in kinetic energy.

Figure 13 shows the temperature and energy changes in the workpiece under different abrasive particle sizes. From Figure 13a, it is evident that with the increase in the abrasive particle sizes, the temperature of the workpiece’s Newtonian layer presents an increasing trend. Under the same displacement distance of the cutting particles, because the larger abrasive particle sizes will act on more workpiece atoms, more cutting heat will be generated. This will enhance the nucleation and activity of dislocations in the workpiece thereby increasing the possibility of internal defect structures appearing. Conversely, a smaller particle size will generate less heat, which will make the temperature of the workpiece more stable. It can be seen from Figure 13b that the potential energy exhibits a gradually increasing trend as the particle size increases. When the abrasive particle sizes are 3.0 nm and 3.4 nm, the potential energy of the workpiece is relatively close. When the abrasive particle size is 2.0 nm, the potential energy of the workpiece is significantly lower than other abrasive particle sizes. This is because the larger size of the abrasive particles increases the processing area, resulting in a greater number of workpiece atoms being compressed and sheared by the abrasive particles. This increases the number of atoms whose lattice structure is destroyed thereby releasing more lattice strain energy, resulting in an increase in the potential energy of the workpiece. As mentioned earlier, the trend of kinetic energy changes is basically the same as the temperature changes. From Figure 13c, it is seen that the atomic kinetic energy of the workpiece increases as the abrasive particle size increases.

**Figure 14:**
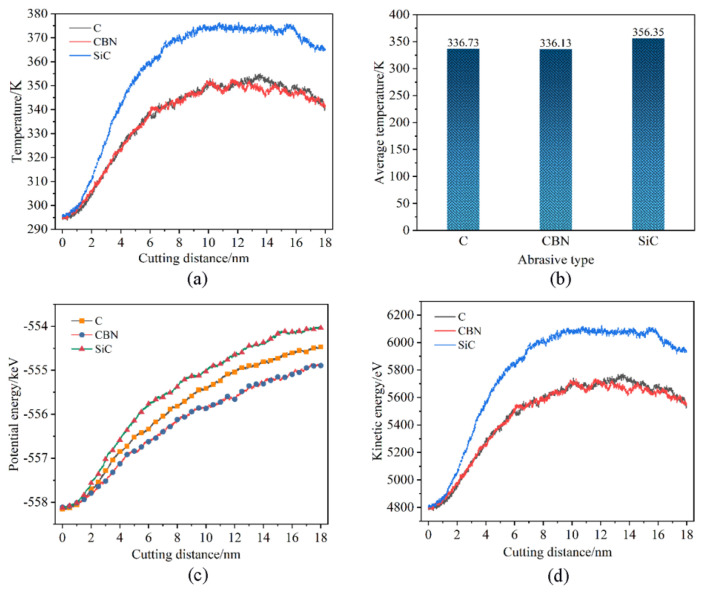
Effects of abrasive materials. (**a**) Variation in Newtonian layer instantaneous temperature, (**b**) variation in Newtonian layer average temperature, (**c**) variation in potential energy, and (**d**) variation in kinetic energy.

The changing law of the temperature and energy changes in the workpiece with the increasing displacement distances of the cutting particles at different abrasive materials is shown in Figure 14. It is seen that the heat generated during the cutting process using SiC abrasive particles is significantly higher compared to that produced by diamond and CBN abrasive particles. In the case of diamond grit and CBN processing, the workpiece temperature is relatively close, but the CBN is slightly low by comparison. As mentioned earlier, when processing γ-TiAl alloy workpieces with SiC abrasive grains, the cutting force fluctuates greatly, which leads to an increase in cutting heat on the workpiece surface, an increase in workpiece surface temperature, and the promotion of lattice deformation as well as defect structure evolution inside the workpiece. Only from the point of view of temperature analysis, when diamond abrasive and CBN abrasive are used, relatively little cutting heat is generated, and relatively little internal dislocation and defect activity in the machining area occur. From Figure 14c, it is seen that with the increase in the displacement distance of the cutting particles, the potential energy of the three abrasive materials shows a gradually increasing trend during processing. However, the range of potential energy changes when using different abrasive materials is different. When using SiC abrasive particles, the potential energy changes greatly, and the increase is most obvious. The potential energy change is relatively small when using CBN abrasive particles. Diamond abrasive particles are somewhere in between. The continuous increase in potential energy is attributed to the release of stored strain energy from the destroyed lattice structure. So, this can be explained as the squeezing and shearing effects of SiC abrasive particles on the workpiece, leading to the destruction of the lattice structure and the generation of higher energy, resulting in greater potential energy conversion. As mentioned earlier, the trend of kinetic energy changes is basically the same as temperature changes. From Figure 14d, it is seen that the kinetic energy of the workpiece atoms during SiC abrasive processing is relatively large, while the diamond abrasive particles and CBN abrasive particles are relatively small and relatively close during processing.

### 3.5. Dislocation Evolution Analysis

In the micro-cutting process, the workpiece undergoes a squeeze–cutting action from the diamond abrasive particles, the workpiece atoms in the cutting area are displaced, and the original lattice structure changes, resulting in dislocations in the workpiece material. Dislocations are the dividing line between the slipped area and the unslip area on the slip surface of the crystal; its existence makes each atom in the crystal shift from its original equilibrium position in its natural state. When the position of the dislocation line changes, the configuration of the atoms in the crystal also changes. Therefore, the evolution of internal dislocations in the workpiece during the machining process is of great significance for the machining effect. In the figure, purple, green, yellow, red, blue, and light blue represent Lomer–Cottrell dislocations, Shockley dislocations, Hirth dislocations, other dislocations, perfect dislocations, and Frank dislocations, respectively. 

**Figure 15:**
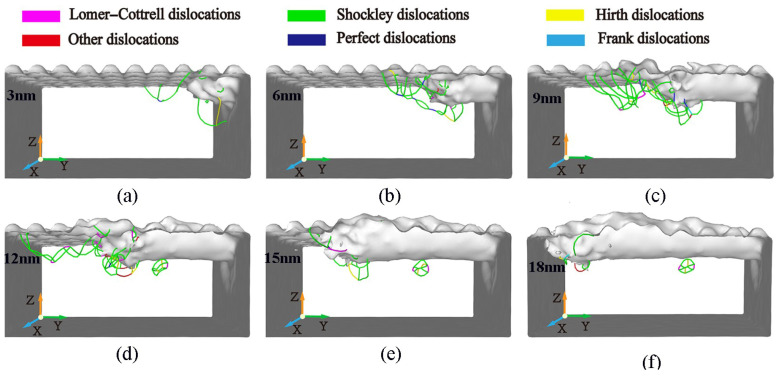
Evolution of dislocations at different displacement distance of cutting particles. Displacement distances of cutting particles are (**a**) 3 nm, (**b**) 6 nm, (**c**) 9 nm, (**d**) 12 nm, (**e**) 15 nm, and (**f**) 18 nm.

The distribution of dislocation defects in the workpiece with the increasing displacement distances of the cutting particles is shown in Figure 15. It can be observed that the dislocations mainly nucleate in the shear slip zone and the subsurface area and some dislocations are in contact with each other and intertwined in a certain shape when they are squeezed and sheared by abrasive particles. Each dislocation will slip and expand along its Burgers vector. Some dislocations will extend to the free surface of the workpiece and then annihilate, and some dislocations will evolve into other dislocation defects through dislocation reactions. From Figure 15a, it is seen that when the displacement distance of the cutting particles is smaller, the number and types of dislocations inside the workpiece are relatively fewer. This is because the material is still in the initial cutting stage, and at this point, the energy generated by cutting is insufficient to nucleate more dislocations. Shockley incomplete dislocations are generated by the decomposition of total dislocations. This is because the total dislocation on the (111) plane possesses high energy and is extremely unstable, and it will automatically decompose into two incomplete dislocations. Shockley dislocation can only make a sliding motion on the (111) plane where it is located and cannot climb from one plane to another. Perfect total dislocations and Hirth dislocations are generally produced through dislocation reactions under the influence of stress and energy. When the displacement distance of the cutting particles reaches 6 nm, there is a rapid increase in both the number and types of dislocations. The total length of the dislocation line reaches 491.919 Å, which is attributed to the combined effect of stress and energy. Shockley dislocations on different (111) planes can generate Lomer–Cottrell dislocations through dislocation reactions. It is also a kind of pressure rod dislocation, and its existence will also hinder the movement of Shockley dislocation. When the displacement distance of cutting particles reaches 9 nm and 12 nm, compared to any other stage, the number and types of dislocations is higher. The total length of the dislocation line measures 921.334 Å and 866.715 Å, respectively. Frank dislocation occurs when the displacement distance of the cutting particles is 9 nm; this kind of dislocation is also called partial dislocation. Different from Shockley’s incomplete dislocation, it can only perform a climbing motion on the (111) plane, but not a sliding motion. When the displacement distance of the cutting particles increases, the number and types of dislocations are greatly reduced and gradually tend toward a stable state. From Figure 15f, it can be seen that there are still some dislocations on the subsurface of the workpiece after processing.

The evolution of dislocations directly impacts the surface quality and material performance of the workpiece. As dislocation density increases, the processed surface exhibits greater lattice distortions, leading to higher surface roughness. In Figure 15, it is evident that when the displacement distance reaches 6 nm or more, the rapid rise in dislocation density correlates with subsurface deformation, which adversely affects surface quality. Dislocations extend to the surface leave lattice distortions, contributing to rougher surfaces and affecting the functional qualities of the workpiece. Beyond 12 nm, the stabilization of dislocation density reduces surface irregularities, achieving a better balance between material removal and surface quality.

**Figure 16:**
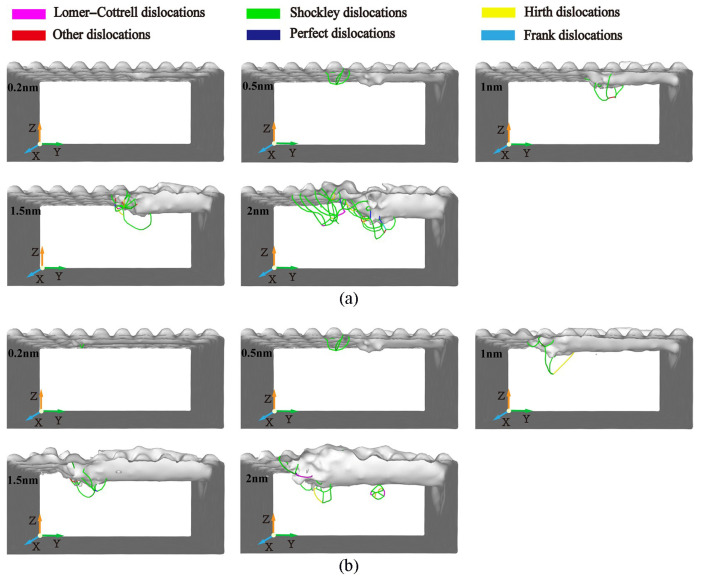
Dislocation distributions in workpiece during machining with abrasive particle cutting depths of 0.2 nm, 0.5 nm, 1 nm, 1.5 nm, and 2 nm. (Displacement distance of cutting particles is (**a**) 9 nm, (**b**) 15 nm).

Figure 16 shows the comparison of dislocation changes at different cutting depths. There is almost no dislocation nucleation inside the workpiece when the cutting depth is 0.2 nm. As the cutting depth increases, more dislocations can be observed in the cutting area, and the dislocation total length and density also increase. As the cutting depth increases, the cutting force and temperature both increase, promoting the nucleation and movement of dislocations. The nucleation, motion, and annihilation of dislocations can cause internal defect structures in workpieces. Therefore, it can be understood that when the cutting depth increases, the workpiece area and number of atoms in contact with the abrasive particles during the machining process will increase, resulting in an increase in the abrasive cutting force and the workpiece temperature. Under the influence of energy and stress, the dislocation activity is more frequent, which eventually leads to a large number of defective structures in the subsurface area of the workpiece. The relationship between cutting depth and dislocation activity also directly affects the final surface quality of the workpiece. According to the analysis in Figure 16, a larger cutting depth leads to a significant increase in dislocation density and activity, resulting in increased lattice distortion and surface roughness. This figure emphasizes how the variation in dislocations increases sharply with the increase in cutting depth, which is related to the formation of defect structures in dislocation regions. On the contrary, at 0.2 nm, a smaller cutting depth will produce fewer dislocations thereby minimizing subsurface defect structures and improving surface smoothness to the greatest extent possible.

**Figure 17:**
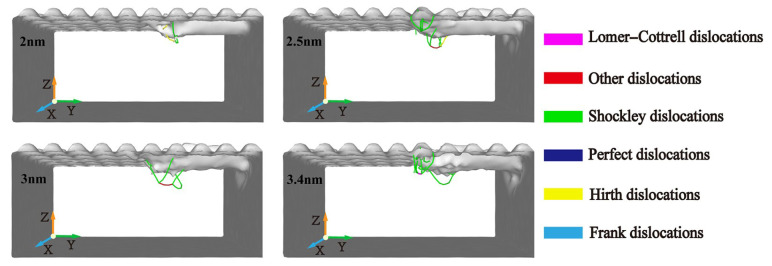
Dislocation distributions in workpiece during machining with abrasive particle sizes of 2 nm, 2.5 nm, 3 nm, and 3.4 nm. (Displacement distance of cutting particles is 9 nm).

The distribution of dislocation defects in the workpiece with the increasing displacement distances of the cutting particles at varying abrasive particle sizes is shown in Figure 17. When selecting different abrasive particle sizes to process the workpiece, the types and numbers of internal dislocations in the workpiece are also different. It can be seen that when the abrasive particle size is smaller, the number of internal dislocations in the workpiece is smaller, and the length of the dislocations is shorter. When the abrasive particle size increases, the cutting force of the abrasive particles on the workpiece increases, generating more energy and cutting heat thereby promoting the nucleation of dislocations within the workpiece. Therefore, various types of dislocations appear during the processing of different abrasive particle sizes, and the lengths and sizes of the dislocations are different. It can be considered that smaller abrasive particle sizes can reduce the nucleation of dislocations, reduce the possibility of defective structures, and thereby improve the subsurface quality. 

The data in Figure 17 also reveal that larger abrasive particle sizes exacerbate subsurface deformation, which negatively impacts the mechanical properties of the workpiece. The increased energy and cutting heat generated with larger particles contribute to excessive lattice distortions and stress concentrations, which may lead to reduced fatigue resistance and durability. On the other hand, smaller abrasive particles, while slightly lowering material removal rates, ensure fewer dislocations and more uniform subsurface quality thereby enhancing the overall mechanical performance of the workpiece.

**Figure 18:**
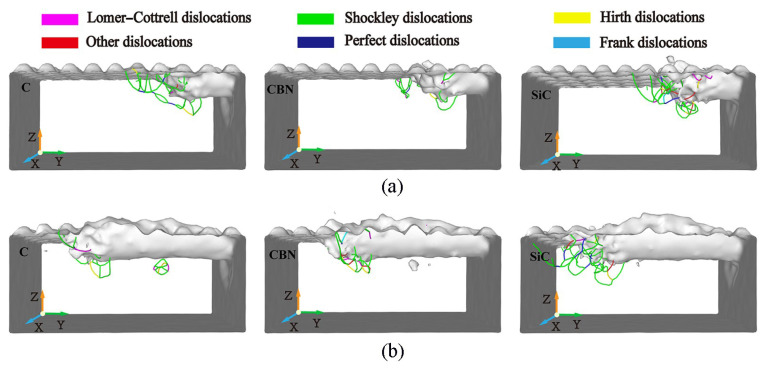
Dislocation distributions in workpiece during machining with abrasive materials of diamond (C), cubic boron nitride (CBN), and silicon carbide (SiC). (Displacement distances of cutting particles are (**a**) 6 nm, (**b**) 15 nm).

The distribution of dislocation defects in the workpiece with the increasing displacement distances of the cutting particles at different abrasive materials is shown in Figure 18. From Figure 18, it can be seen that under the squeezing and shearing action of the abrasive particles, no matter what kind of abrasive material is used for processing, dislocations will always nucleate and slip in the shear slip zone of the workpiece. The types and numbers of dislocations are very different when different abrasive particles are used for processing, resulting in very different dislocation densities. When using CBN abrasive particles for processing, the number of dislocations on the surface of the workpiece before and after processing is relatively small, and the activity and density of dislocations are also low. However, when using SiC abrasive particles for processing, there are many dislocations in both the pre- and post-stages, and the dislocation density is also relatively high. When using diamond abrasive particles for processing, the dislocation density is relatively large in the initial stage of processing and relatively small in the later stage. As mentioned above, the nucleation, movement, and annihilation of dislocations led to many defective structures in the shear slip zone and subsurface of the workpiece. Therefore, when the abrasive material is processed by CBN abrasive particles, the internal dislocation activity is less, and the internal defect structure is less. This is beneficial for improving the subsurface quality of the workpiece.

The difference in dislocation activity and density caused by different abrasives directly affects the mechanical properties and surface quality of the workpiece. According to the analysis in Figure 18, the low dislocation density and reduced defect structure generated by using CBN abrasive contribute to improving subsurface integrity and enhancing fatigue resistance and overall durability. In contrast, the high dislocation density observed using SiC abrasives can lead to lattice distortion and increased stress concentration, which may reduce the fatigue life of the workpiece. Diamond abrasives exhibit mixed behavior, where initial high dislocation density helps to effectively remove materials, but later stabilization contributes to achieving better surface quality.

## 4. Conclusions

In this study, a molecular model of a single crystal γ-TiAl alloy micro-cutting with abrasive particles in a fluid medium is established to study material removal, cutting force changes, temperature and energy changes, and dislocation changes during micro-cutting. The influence of different machining parameters (depth of cutting, abrasive particle sizes, and abrasive materials) on the micro-cutting process was investigated. Based on the above analysis, the following conclusions can be drawn:

1. Under the squeezing and shearing action of abrasive particles, some of the workpiece atoms leave their initial positions hence the workpiece atoms are displaced, and dislocations will occur between workpiece atoms that are displaced in different directions. Part of the workpiece atoms move to the front and above the abrasive particles, a large amount of them accumulate in the front end of the abrasive particles to form chips, and some of them move to the front and below the abrasive particles, resulting in a processed surface.
2. In the micro-cutting of a single crystal γ-TiAl alloy, a larger cutting depth leads to severe subsurface deformation due to increased cutting forces, which destroy more atomic lattice structures and generate more heat and energy, promoting dislocation nucleation. Although setting a smaller cutting depth will reduce the number of atoms removed from the material, it will also greatly reduce the degree of deformation of the subsurface of the workpiece.
3. In the abrasive flow machining of a single crystal γ-TiAl alloy, larger abrasive particle sizes improve material removal efficiency but cause severe subsurface deformation by enlarging the processing area, displacing more atoms, destroying lattice structures, and generating heat that promotes dislocation nucleation. Although choosing a smaller abrasive particle size will reduce the material removal efficiency, it will also reduce the nucleation of dislocations and obtain a better-processed surface.
4. Different abrasive materials have different processing effects. When choosing a CBN abrasive micro-cutting single crystal γ-TiAl alloy, the fluctuation of cutting force is small, and the cutting heat and energy generated by abrasive micro-cutting are less, which suppresses the nucleation of internal dislocations in the workpiece and reduces the possibility of defect structures appearing. Therefore, CBN abrasive particles are beneficial to the precision machining of a single crystal γ-TiAl alloy abrasive flow and can effectively replace extremely expensive diamond abrasive particles.
5. The influence of cutting depth, abrasive particle size, and abrasive type on machining performance has a significant coupling effect. Greater cutting depth and abrasive grain size typically improve material removal efficiency, but may lead to higher cutting force fluctuations, thermal effects, and subsurface deformation; smaller abrasive particle size and shallow cutting depth can improve surface quality and reduce subsurface damage. In addition, the mechanical properties of abrasive types such as diamond, CBN, and SiC determine their machining efficiency and stability under different parameter combinations. By optimizing the combination between cutting depth, abrasive particle size, and abrasive type, a balance between high efficiency and high-quality machining can be achieved.

## Figures and Tables

**Table 1:** Simulation conditions and parameters of MD simulation.

Analog Parameter	Simulation Parameters
Workpiece size	120.03 Å × 220.055 Å × 112.887 Å
Fluid medium	N-dodecane
Time step	1 fs
The initial temperature	293 K
Cutting speed	100 m/s
Displacement distance of cutting particles	18 nm
Cutting crystal orientation	(001)[0–10]
Cutting depth	0.2 nm, 0.5 nm, 1 nm, 1.5 nm, 2 nm
Abrasive particle size	2 nm, 2.5 nm, 3 nm, 3.4 nm
Abrasive material	Diamond, Cubic SiC, Cubic CBN

**Table 2:** Morse potential function parameters [33,34,35].

	Ti-C	Ti-B	Ti-N	Ti-Si	Al-B	Al-C	Al-N	Al-Si
D (eV)	0.9820	0.9646	1.3326	1.2801	0.8988	0.2800	1.2417	1.1927
α (Å^−1^)	2.2830	1.1773	1.7861	0.8471	1.2116	2.7800	1.8381	1.0375
r_0_ (Å)	1.8920	1.8126	1.9148	2.965	1.9231	2.2000	2.0316	3.1459

**Table 3:** L-J potential function parameters [37,38].

	σ (Å)	ε (eV)	Mass (g/mol)
CH_2_	3.95	0.0039639	14.0272
CH_3_	3.75	0.0084449	15.0351
Ti	4.149	0.003742	47.8670
Al	2.620	0.392000	26.9815
C	3.369	0.002635	12.0110
B	3.453	0.004116	10.8110
N	3.365	0.006281	14.0067
Si	3.826	0.017500	28.0850

## Data Availability

The original contributions presented in the study are included in the article, further inquiries can be directed to the corresponding authors.
